# Characterization of Unilateral Adrenal Incidentalomas: Hormonal Analysis, Computed Tomography, and Magnetic Resonance Imaging Correlation

**DOI:** 10.7759/cureus.100542

**Published:** 2025-12-31

**Authors:** Ayoub Gasmi, Ach Taieb, Fatma Hattab, Aycha Ghachem, Nassim Ben Haj Slama, Imen Halloul, Wiem Saafi, Hajer Marzouk, Hamza Elfekih, Ghada Saad, Yosra Hasni, Houda Mhabrech

**Affiliations:** 1 Department of Radiology, University Hospital of Farhat Hached Sousse, Sousse, TUN; 2 Laboratory of Exercise Physiology and Pathophysiology, University Hospital of Farhat Hached Sousse, Sousse, TUN; 3 Diabetology and Endocrinology, University Hospital of Farhat Hached Sousse, Sousse, TUN; 4 Endocrinology, University Hospital of Farhat Hached Sousse, Sousse, TUN; 5 Endocrinology, Ibn El Jazzar Kairouan University Hospital, Sousse, TUN; 6 Diabetology and Endocrinology, University of Sousse, Faculty of Medicine of Sousse, Sousse, TUN

**Keywords:** adenoma, adrenal cortical carcinoma, adrenal gland, ct, mri, pheochromocytoma

## Abstract

Introduction: Adrenal incidentalomas are adrenal masses detected on imaging performed for reasons other than suspected adrenal disease. Although most adrenal incidentalomas are nonfunctioning adenomas, some require treatment, particularly hormonally active or malignant lesions.

Objective: The purpose of this study was to analyze the clinico-hormonal and radiological characteristics of adrenal incidentalomas, to determine radiological features predictive of malignancy, and to compare nonfunctioning and cortisol-secreting adenomas in terms of radiological features.

Methods: This study involved 153 adult patients diagnosed with adrenal incidentaloma between 2015 and 2023. Clinical, hormonal, and imaging data were collected for all participants.

Results: The mean age of patients was 55 ± 13 years. Autonomous cortisol secretion was the most common hormonal activity, observed in 17% of cases, while 73.85% of patients had non-functional tumors. The most frequent diagnosis was adenoma, identified in 80.4% of patients. Adrenocortical carcinoma and pheochromocytoma were each diagnosed in 3.9% of patients. The mean size was significantly greater in adrenocortical carcinoma cases than in lipid-poor adenomas (61 ± 27.07 mm vs. 26.97 ± 11.85 mm; p = 0.027). The mean non-contrast CT density of adrenocortical carcinoma was 29.7 Hounsfield units, with a range of 18 to 41. Furthermore, cortisol-secreting adenomas were significantly larger than non-secreting adenomas and were more often lipid-poor. Contralateral adrenal atrophy and hepatic steatosis were significantly more prevalent in cortisol-secreting adenomas compared to non-secreting adenomas: (26.1% [n = 6] vs. 1.1% [n = 1]; p < 10⁻³) and (47.8% [n = 11] vs. 18.1% [n = 17]; p = 0.003), respectively.

Conclusions: We examined the clinical, radiological, and hormonal profiles of patients with adrenal incidentalomas, finding results consistent with existing literature. The main etiology in our series was a non-functioning adrenal adenoma. Autonomous cortisol secretion is the most prevalent hormonal activity. Imaging played a key role not only in distinguishing benign from malignant lesions but also in identifying features suggestive of cortisol secretion.

## Introduction

Adrenal incidentalomas (AIs) are adrenal masses discovered on imaging performed for reasons other than suspected adrenal disease, measuring at least 1 cm in short-axis diameter [1-3]. This definition excludes radiological evaluations in patients with genetic syndromes predisposing them to adrenal tumors, as well as abdominal imaging performed for the staging of an extra-adrenal tumor.

The incidence of AIs is increasing due to the widespread utilization of radiographic imaging techniques. AIs are found in approximately 5% of patients who undergo abdominal computed tomography (CT) examinations [3]. 

Thorough clinical assessment, including a detailed medical history, imaging evaluation of adrenal tumor characteristics, and biochemical testing to rule out hormonal excess, can help clinicians identify the rare cases that carry a significant health risk.

Although most AIs are nonfunctional and benign [2], a subset exhibits secretory activity, known as functioning adenomas. Diagnostic evaluation for adrenal hormone excess involves measuring plasma or urinary metanephrines, performing the 1-mg overnight dexamethasone suppression test, and, in cases with hypertension or hypokalemia, assessing aldosterone and plasma renin activity [4].

The primary objective of imaging examinations is to differentiate adrenal adenomas from other adrenal lesions that may necessitate further diagnostic evaluation and to identify radiological features indicative of potential malignancy. Lesion size has been regarded as an indicator of malignancy, given that most adrenocortical carcinomas (ACCs) are significantly larger than adenomas at the time of diagnosis. However, size alone has limited specificity for distinguishing benign from malignant lesions, as ACCs may also present as relatively small tumors in their early stages [5]. Beyond size, additional imaging features suggestive of malignancy include a heterogeneous appearance with areas of necrosis or hemorrhage, as well as signs of invasion into adjacent structures.

Certain imaging characteristics, such as adrenal nodule size and density, have been shown to provide important clues for assessing the likelihood of autonomous cortisol secretion (ACS) [6]. Some extra-adrenal CT findings may also be linked to ACS.

The purpose of this study was to analyze the clinico-hormonal and radiological characteristics of AIs to identify key features for determining malignancy and compare nonfunctioning and cortisol-secreting adenomas in terms of radiological features.

## Materials and methods

This was a retrospective, single-center, cross-sectional study conducted at the Farhat Hached University Hospital in Sousse, Tunisia. Patients were identified from January 1, 2015, to December 31, 2023.

Study population

The sample size was calculated using the standard formula for prevalence studies:

n = z² × p (1 - p) / i²

Where n is the required sample size and z is the z-score corresponding to the desired confidence level (for 95% confidence, z = 1.96). p is the estimated prevalence of AIs [3], reported to range from 0.5% to 4.2% in radiological studies; i is the margin of error, set at 5%.

The calculated sample size was n = 61. This value was deemed statistically representative of the target population. However, anticipating a possible increase in the prevalence of AIs, the sample size was increased. The final study cohort included n = 153 patients. We included 153 adult patients who were diagnosed with AIs, referred to the department of endocrinology and diabetology at Farhat Hached University Hospital in Sousse, and who underwent biochemical and endocrine evaluation. Imaging procedures were performed for gastrointestinal or urogenital symptoms, general checkups, or preoperative evaluation.

The inclusion criteria were adult patients with adrenal masses detected on imaging with complete medical records focusing on both clinical and paraclinical aspects: Patients diagnosed with a unilateral adrenal incidentaloma (≥1 cm in short-axis diameter) on cross-sectional imaging (CT or MRI) and for whom a complete medical record, including detailed clinical history, full hormonal workup, and radiological characterization, was available.

Exclusion criteria included patients followed for a known neoplastic condition, those with bilateral AIs, individuals with a history of previous adrenal surgery, or those with a personal or family history of genetic predisposition to adrenal pathologies (e.g., multiple endocrine neoplasia type 1, Li-Fraumeni syndrome). Patients with bilateral AIs were excluded due to their distinct etiological profile, often involving genetic syndromes, hyperplasia, or systemic diseases, which necessitate a different diagnostic and management approach compared to unilateral incidentalomas. This exclusion ensured a more homogeneous cohort for analyzing the specific radiological and hormonal correlations central to our study objectives.

We collected the following data for all patients: age, gender, body mass index, blood pressure, palpitations, sweating, polydipsia or polyuria, facial ruddiness, and moon facies. Hypertension was defined as follows: a systolic blood pressure ≥140 mmHg and/or a diastolic blood pressure of ≥90 mmHg [7]. 

Diagnostic criteria

Pheochromocytoma (PCC) was diagnosed based on the combination of clinical signs and symptoms, elevated plasma catecholamine levels, elevated 24-h urinary catecholamine metabolites, and a positive 123I-metaiodobenzylguanidine scintigraphy result. The diagnosis of autonomous cortisol secretion (ACS) requires the presence of an AI with or without cushingoid features and the lack of serum cortisol suppression following 1-mg dexamethasone suppression tests (> à 50 nmol/L). The diagnosis of primary aldosteronism (PA) was based on the simultaneous measurement of plasma aldosterone and renin activity to calculate the aldosterone-to-renin ratio, with values >20 considered positive.

Image analysis

The initial radiological examinations conducted included CT and magnetic resonance imaging (MRI). For each patient, we documented tumor location and maximal axial diameter, noting the presence of calcifications, central necrosis, obliteration of the peritumoral fat planes, and invasion into surrounding structures (Table 1).

**Table 1:** Radiological features assessed in adrenal incidentalomas CT: computed tomography; MRI: magnetic resonance imaging; ACC: adrenocortical carcinoma; PCC: pheochromocytoma.

Radiological Feature	Imaging Characteristics (CT/MRI)	Clinical/Diagnostic Significance
Calcifications	Punctate, rim-like, or coarse high-attenuation foci (CT); low signal on all MRI sequences.	Non-specific; seen in benign (adenoma, cyst) and malignant (ACC, PCC) lesions.
Central Necrosis	Non-enhancing central area with low attenuation (CT)/high T2 signal (MRI).	Suggests rapid growth; common in ACC, PCC, large adenomas; indicates possible malignancy.
Obliteration of Peritumoral Fat Planes	Loss of fat interface between tumor and adjacent structures; stranding or infiltration.	May suggest local invasion if associated with malignancy; can also occur due to inflammation.
Invasion into Surrounding Structures	Irregular tumor margins extending into adjacent organs/vessels; vascular thrombus or encasement.	Highly suggestive of malignancy (ACC); critical for staging and surgical planning.

For calculating the washout, we recorded each nodule’s attenuation in Hounsfield units (HU) during the non‑contrast phase, the portal venous phase (approximately 60-70 seconds post‑contrast bolus), and the delayed phase (typically 15 minutes post‑bolus). On MRI, signal intensity was measured on axial in‑phase and opposed‑phase T1‑weighted gradient‑echo images to calculate the adrenal signal intensity index, with values exceeding 16.5% strongly suggesting an adenoma diagnosis. Additional sequences, including T2‑weighted, fat‑saturated post‑contrast T1‑weighted, and coronal/sagittal reformats, were systematically reviewed to evaluate features such as heterogeneity, necrosis, and potential invasion into adjacent structures or vessels.

The adrenal signal intensity index was evaluated, with threshold values exceeding 16.5% strongly suggesting an adenoma diagnosis [6]. In addition to the adrenal lesion, we examined potential atrophy of the contralateral adrenal gland.

Statistical analysis

Statistical analysis was performed using IBM Corp. Released 2017. IBM SPSS Statistics for Windows, Version 23. Armonk, NY: IBM Corp. Categorical variables are presented as numbers or percentages, while normally distributed continuous variables are expressed as mean ± standard deviation. Continuous variables were pairwise compared between groups by using the student’s t-test. Categorical variables were analyzed by using the chi-squared test. A p-value < 0.05 denoted the presence of a statistically significant difference.

## Results

Clinical and hormonal evaluation

The mean age was 55 ± 13 years. Most of the patients were female (61.4%). Hypertension was found to be the most common comorbidity, followed by diabetes mellitus. Prevalence rates for hypertension, diabetes mellitus, and hyperlipidemia were 41.2%, 30.1%, and 8.5%, respectively. Patients were categorized into nonfunctioning (73.9%) and functioning tumor (26.1%) groups based on hormonal workup assessments. Of the patients with functioning tumors, 5.2% of cases had PA, 17% of cases had ACS, and 3.92% of cases had PCC. The Cushing syndrome included 23 adenomas, two ACCs, and one myelolipoma. The prevalence of hypertension was significantly higher in patients with PA, affecting 85.7% of them, followed by patients with ACS, affecting 61.5%. Glucose intolerance was more frequently observed in patients with ACS and PA (57.7% and 57.1%, respectively) (Table 2). 

**Table 2:** Main clinical and biological characteristics of each adrenal incidentaloma subtype BMI: body mass index; ACS: autonomous cortisol secretion; PA: primary aldosteronism; PCC: pheochromocytoma. Data are expressed as mean ± SD and N (%).

Parameter	Non-secreting (n=115)	ACS (n=26)	PA (n=8)	PCC (n=6)
Age (years)	56.4 ± 9	57 ± 11	56 ± 16	45 ± 10
BMI (kg/m²)	28.1 ± 5.7	28 ± 6	30.5 ± 6	26.9 ± 3.5
Hypertension	34 (29.5%)	16 (61.5%)	6 (85.7%)	3 (50%)
Potassium (mEq/L)	4.1 ± 0.3	3.6 ± 0.5	3.4 ± 0.5	4.2 ± 0.7
Dyslipidemia	25 (22%)	9 (34.6%)	3 (43%)	2 (33%)
Glucose intolerance	47 (41%)	15 (57.7%)	4 (57.1%)	1 (16.7%)

Radiological evaluation

The radiological evaluation included CT scans in 153 patients and MRI scans in 24 patients. Of the 153 patients, 11.11% underwent surgery. The indications for surgery included tumor size, imaging features suggestive of malignancy, and hormone secretion (Table 3).

**Table 3:** Final diagnoses of adrenal incidentalomas in our study population Values expressed as N (%).

Diagnosis	Number (n)	Percentage (%)
Adrenocortical adenoma	123	80.2
Adrenocortical carcinoma	6	3.9
Pheochromocytoma	6	3.9
Adrenal hemangioma	2	1.4
Adrenal cyst	3	2.0
Adrenal metastasis	2	1.4
Myelolipoma	11	7.2

Radiological evaluation revealed that the mean tumor size was significantly greater in ACC cases than in lipid-poor adenomas (61 ± 27.07 mm vs. 26.97 ± 11.85 mm; p = 0.027). Among the six ACCs, only one measured less than 4 cm. PCC also tended to be larger, with eight cases exceeding 5 cm. In contrast, benign lesions were predominantly smaller, with only four myelolipomas, five adenomas, and two cysts exceeding 4 cm (Table 4).

**Table 4:** Outcomes of different size thresholds in patients with adrenal incidentalomas ACCs: Adrenocortical carcinomas; PCC: Pheochromocytomas. Variables are expressed as (N,%).

Size	Malignant Lesions (n,%) (n subtypes)	Benign Lesions (n, %) (n subtypes)
> 3 cm	n=7 (4.6%) (6 ACC + 1 metastasis)	n=38 (24.8%) (5 myelolipomas, 30 adenomas, 2 cysts, 1 hemangioma)
> 4 cm	n=6 (3.9%) (5 ACC + 1 metastasis)	n=11 (7.2%) (4 myelolipomas, 5 adenomas, 2 cysts)
> 5 cm	n= 4 (2.6%) (3 ACC + 1 metastasis)	n=7 (4.6%) (2 myelolipomas, 3 adenomas, 2 cysts)
> 6 cm	n=3 ACC (2%)	n=3 (2%) (1 myelolipoma, 2 adenomas)

CT scan analysis of tumor density revealed that 62.7% of lesions had a non-contrast CT density (NCD) of 10 Hounsfield units (HU) or less. The mean NCD of ACC was 29.7 HU, ranging from 18 HU to 41 HU. Overall, 62.7% of lesions exhibited a density of less than 10 HU, indicating a lipid-rich adenoma. Among the patients who had a CT scan with washout calculation, 35.3% of patients had a washout that was not significant, 54.3% of whom were confirmed as adenomas based on histological findings or MRI. Fat density was present in 11 cases of myelolipomas and in one adrenal adenoma with foci of myelolipomatous degeneration.

Calcifications were identified in 11.1% of the CT scans performed. These were found in 10 adenomas, two ACCs, two cysts, two myelolipomas, and one PCC. Central necrosis, adjacent fat densification, and invasion into surrounding structures were observed in 6.5%, 7.2%, and 2% of cases, respectively. Among the six ACCs, five showed central necrosis. Necrosis was also present in three PCCs, one hemangioma, and one large adenoma. In our series, organ compression, most commonly involving the ipsilateral kidney, was observed in 19 cases, including ACCs and PCCs, as well as adenomas, myelolipomas, and adrenal cysts. Three lesions were responsible for invading adjacent structures, all of which were ACCs. Among them, two showed vascular invasion (Table 5).

**Table 5:** Comparison of radiological features between adrenocortical carcinoma and lipid-poor adenomas Data expressed as mean ± SD and N (%). Student’s t-test was used for the comparison of tumor size. The chi-square test was used for size ≥40 mm, calcifications, necrosis, and adjacent fat densification. Statistical significance was defined as p < 0.05.

Variable	ACC (n=6)	Lipid-poor adenoma (n=48)	Test statistic	p-value
Tumor size (mm)	61 ± 27.07	26.97 ± 11.86	t = 3.043	
Size ≥ 40 mm	5/6 (83.3%)	5/48 (10.4%)	χ² = 18.793	<10⁻³
Calcifications	2/6 (33.3%)	5/48 (10.4%)	χ² = 2.483	0.115
Necrosis	4/6 (66.7%)	1/48 (2.1%)	χ² = 26.477	< 10⁻³
Adjacent fat stranding	4/6 (66.7%)	1/48 (2.1%)	χ² = 26.477	< 10⁻³

Cortisol-secreting adenomas were significantly larger than non-secreting adenomas (27.55 ± 11.7 mm vs. 20.5 ± 9.3 mm; p = 10⁻³) and were more often lipid-poor (72.4% [n = 16] vs. 28.7% [n = 27]; p < 10⁻³). Contralateral adrenal atrophy and hepatic steatosis were significantly more prevalent in cortisol-secreting adenomas compared to non-secreting adenomas: 26.1% [n = 6] vs. 1.1% [n = 1]; p < 10⁻³ and 47.8% [n = 11] vs. 18.1% [n = 17]; p = 0.003, respectively (Table 6).

**Table 6:** Comparison analysis between non-functioning adenomas and cortisol-secreting adenomas Data expressed as mean ± SD and N (%). Student’s t-test was used for comparison of tumor size. The chi-square test was used for lipid-poor adenoma, contralateral adrenal atrophy, and hepatic steatosis. Statistical significance was defined as p < 0.05.

Variable	Cortisol-secreting (n=23)	Non-secreting (n=94)	Test statistic	p-value
Tumor size (mm)	27.55 ± 11.7	20.5 ± 9.3	t = 2.689	<10^-3^
Lipid-poor adenoma	16/23 (72.4%)	27/94 (28.7%)	χ² = 13.260	< 10^-3^
Contralateral adrenal atrophy	6/23 (26.1%)	1/94 (1.1%)	χ² = 20.570	< 10^-3^
Hepatic steatosis	11/23 (47.8%)	17/94 (18.1%)	χ² = 8.979	0.003

## Discussion

The literature is inconsistent regarding comorbidities in patients with AI. In our series, 30.1% of patients had diabetes and 41.2% had hypertension. Based on these findings, diabetes and hypertension appear to be more prevalent among patients with AIs than in the general population. Previous studies have suggested that diabetes mellitus and hypertension are more common in AI patients than in the general population [1,8]. However, this association remains controversial and is largely influenced by patient age. In our study, the prevalence of hypertension was markedly higher in the group with PA, followed by the group with ACS. Several studies have demonstrated an association between ACS and the development of hypertension, although hypertension remains a well-established clinical feature of PA [9].

Our analysis revealed that the majority of AI were non-functional (73.88%). According to a recent study on the management of AI, the prevalence of non-functional lesions ranges from 40% to 70% [10]. In our series, ACS was the most frequently observed hormonal abnormality, present in 17% of cases. The reported prevalence of ACS among AI ranges between 20% and 50% [10]. This variability may be attributed to differences in exclusion criteria and the use of varying diagnostic thresholds. Higher prevalence rates have been reported in studies that included mild forms of ACS, defined by a post-dexamethasone cortisol level greater than 50 nmol/L, without additional stratification.

In our cohort, PA was identified in 5.2% of cases. Mantero et al. and Kim et al. reported PA prevalence rates of 1.6% and 4.6%, respectively [11,12].In Japan, Tabuchi et al. reported a higher prevalence of 10.5%, which may be explained by the use of a lower aldosterone-to-renin ratio threshold compared to higher thresholds used in other countries [2]. PCC was diagnosed in 3.92% of cases. This finding is consistent with reports by Mantero et al., Kim et al., and Tabuchi et al., who described prevalence rates of 4.2%, 7.2%, and 4.6%, respectively [2,11,12]. In contrast, Arac et al. reported a lower prevalence, which may be due to their exclusive use of urinary assays for the diagnosis of PCC, potentially leading to underdiagnosis [1]. AI requires a thorough radiological assessment to avoid both overdiagnosis and underdiagnosis. The establishment of standardized criteria is essential for effective management, although these criteria remain a subject of debate due to the ongoing evolution of scientific knowledge.

Attenuation value at CT is a feature that indicates lipid content in an adrenal adenoma. NCD <10 HU are diagnostic indicators of a lipid-rich adenoma with a sensitivity of 71% and a specificity of 98% [13]. Overall, 96 AIs exhibited a density of less than 10 HU, indicating a lipid-rich adenoma. Conversely, when the density exceeded 10 HU, contrast-enhanced CT or MRI was used to confirm the benign nature of the masses. Notably, none of the PCCs exhibited an NCD below 10 HU. Recent studies have consistently shown that the possibility that an adrenal tumor with HU < 10 in unenhanced CT is a PCC is close to zero [10].

In our observations, the washout was not significant in 35 lesions, of which 19 were confirmed to be adenomas based on histological evidence or MRI. Other pathologies included benign lesions (hemangiomas), PCC, and malignant lesions (metastases and ACC). These results highlight the fact that washout criteria are complementary but not exclusive and should be considered in conjunction with other diagnostic parameters such as heterogeneity and necrosis. Calcifications can be found in both benign and malignant lesions, and their presence alone is not sufficient to make a definitive diagnosis. They may be primary in calcifying diseases such as hemangiomas and cysts or may occur secondarily due to hemorrhage or tumor necrosis. The presence of macroscopic fat is a distinctive radiological feature for the diagnosis of myelolipoma. However, the detection of macroscopic fat on radiological examinations does not completely rule out malignancy, especially when the fat content is less than 5% [14]. Macroscopic fat has also been described in adrenal adenomas [15].

In our observations, we described one AI with areas of fat attenuation on non-enhanced CT, which was histologically identified as an adrenal adenoma with myelolipomatous degeneration. In addition to their typical imaging characteristics, adrenal adenomas may exhibit various other features such as calcifications, hemorrhage, myelolipomatous degeneration, or cystic changes that complicate the accurate identification of atypical cases. Tumor size is considered a key criterion in most studies for distinguishing benign from malignant adrenal masses [16,17]. However, the optimal threshold value has yet to be clearly established. A study published in 2023 compared the guidelines from eight international societies regarding the size threshold for AI. Most recommend a 4 cm cutoff, although notable discrepancies exist among the different recommendations [17]. In line with previous studies, our results showed that the mean size was significantly greater in ACC cases than in lipid-poor adenomas (61 ± 27.07 mm vs. 26.97 ± 11.85 mm; p = 0.027).

The mean NCD in ACC was 29.7 HU, with a range of 18 to 41. In line with our findings, a retrospective study conducted on 2219 cases of AI showed that the risks of ACC based on NCD were 0%, 0.5%, and 6.3% for lesions with NCD below 10 HU, between 10 and 20 HU, and above 20 HU, respectively [18]. In our series, necrosis was observed not only in malignant lesions (four ACC, three PCC, and one metastasis from a pulmonary carcinoma) but also in two benign lesions (a hemangioma and an adenoma). Necrosis is well established as one of the radiologic features suggestive of malignancy in the assessment of adrenal masses [19,20]. In a study involving 18 ACCs and 41 adenomas, necrosis was identified in all ACCs, regardless of their size, with a positive correlation between tumor diameter and necrotic volume [21]. In an interesting study, Zhang et al. evaluated 251 adrenal masses larger than 5 cm. Among the 34 adenomas, 14 demonstrated necrosis on CT, with a reported mean size of 6.38 ± 1.67 cm [22]. It is plausible to speculate that larger tumor dimensions may result in central ischemia, leading to necrosis.

Compression of adjacent organs can occur even in benign adrenal lesions. It is important to note that this compression does not necessarily indicate aggressive behavior, as the interfaces between the mass and the compressed organ remain intact. In our series, organ compression was observed in 19 cases, including ACCs and PCCs, as well as adenomas, myelolipomas, and adrenal cysts. Large benign adrenal lesions have been reported in the literature with signs of organ compression. These lesions were surgically resected, partly to ensure a definitive diagnosis and partly to prevent potential impact on the compressed organs [23-25].

Three lesions were responsible for invading adjacent structures, all of which were ACCs. Among them, two showed vascular invasion involving the ipsilateral renal vein and the inferior vena cava, respectively. In both cases, contrast-enhanced CT demonstrated intravascular tumor thrombus with heterogeneous enhancement similar to the primary adrenal mass. Detection of vascular invasion in adrenal tumors is crucial for preoperative evaluation and treatment planning, especially in the case of ACCs. Vascular invasion appears as a tumor thrombus or an extension of the mass into the vessel lumen. The frequency of radiologically detected inferior vena cava invasion in ACCs ranges from 9% to 19%, depending on the tumor stage and imaging technique used [26]. MRI is particularly helpful in distinguishing true vascular invasion from simple compression or displacement of the vessels. In our series, obliteration of the peritumoral fat planes was observed in 4 ACCs, 2 PCCs, 2 metastases, 2 adenomas, and 1 hemangioma. Indeed, obliteration of the peritumoral fat planes can occur in both malignant and benign lesions due to various inflammatory or reactive phenomena and should not be systematically considered a sign of malignancy or invasion. However, the preservation of fat planes around the tumor indicates the absence of local invasion [20].

Cortisol-secreting adenomas were significantly larger than non-secreting adenomas (27.55 ± 11.7 mm vs. 20.5 ± 9.3 mm; p = 10⁻³) and were more often lipid-poor (72.4% [n = 16] vs. 28.7% [n = 27]; p < 10⁻³). This observation is consistent with the findings of Nasiroglu Imga et al., who noted that larger AIs were more likely to be functional [27]. Tumor size could therefore be considered a useful predictor of hormonal activity. Additionally, Chambre C et al. demonstrated that ACS is associated with high NCD. In their study, 79% of cortisol-secreting adenomas had an NCD greater than 10 HU [28].

The radiological assessment of AIs should extend beyond the adrenal glands to include a systematic evaluation of organs that could be affected by potential hypercortisolism. Subtle radiological signs can help detect hypercortisolism at an early stage, before more obvious clinical symptoms develop. The assessment of adrenal atrophy can provide additional information by indirectly reflecting the state of cortisol or ACTH secretion in the body. Kim et al. showed that the degree of contralateral adrenal atrophy on CT imaging is correlated with the likelihood of cortisol-secreting adenomas [29]. 

This study has several limitations. First, its retrospective and single-center design may limit the generalizability of the findings. Second, long-term follow-up was not available for all patients, preventing evaluation of tumor evolution and recurrence. Finally, the relatively small number of malignant tumors limits the statistical power for subgroup analyses.

## Conclusions

AI constitutes an entity with various etiologies, which can be serious. We systematically examined the clinical, radiological, and hormonal profiles of patients with AIs in our cohort. The main etiology in our series was non-functioning adrenal adenoma, which is in line with previous reports. Notably, ACS emerged as the most prevalent hormonal activity observed among functional adrenal tumors, further supporting its clinical relevance. Imaging features were found to play a pivotal role in the evaluation of adrenal nodules. These features not only help differentiate benign lesions from malignant tumors but also provide valuable clues for predicting ACS.

In the context of malignancy, tumor size, necrosis, and invasion into adjacent structures were key imaging indicators. On the other hand, cortisol-secreting adenomas are more likely to manifest as relatively large and hyperattenuating masses on CT. MRI, particularly chemical-shift imaging, provided complementary value in characterizing lipid content and clarifying invasive or complex morphology. Together, these radiological tools enable a more comprehensive assessment, guiding the prediction of both functional status and malignant potential in adrenal incidentalomas.
